# Assessment of heavy metals accumulation in agricultural soil, vegetables and associated health risks

**DOI:** 10.1371/journal.pone.0267719

**Published:** 2022-06-16

**Authors:** Nehar Ullah, Maqsood Ur Rehman, Bashir Ahmad, Irshad Ali, Muhammad Younas, Muhammad Sagheer Aslam, Atta-ur Rahman, Ensiyeh Taheri, Ali Fatehizadeh, Mashallah Rezakazemi

**Affiliations:** 1 Department of Chemical Engineering, Faculty of Mechanical, Chemical and Industrial Engineering, University of Engineering & Technology, Peshawar, Pakistan; 2 Department of Plant Protection, Agricultural University, Peshawar, Pakistan; 3 National Institute of Urban Infrastructure and Planning, University of Engineering & Technology, Peshawar, Pakistan; 4 Department of Geography, University of Peshawar, Peshawar, Pakistan; 5 Environment Research Center, Research Institute for Primordial Prevention of Non-communicable Disease, Isfahan University of Medical Sciences, Isfahan, Iran; 6 Department of Environmental Health Engineering, School of Health, Isfahan University of Medical Sciences, Isfahan, Iran; 7 Faculty of Chemical and Materials Engineering, Shahrood University of Technology, Shahrood, Iran; Ghazi University, PAKISTAN

## Abstract

Industrialization plays a vital role in the development of a country’s economy. However, it also adversely affects the environment by discharging various unwanted and harmful substances such as heavy metals into the surface and subsurface aquifers. The current research work investigates the identification, characterization, and evaluation of specific heavy metals in industrial wastewater (IWW) and different composite samples of soil and vegetables (onion, pumpkin, lady finger, and green pepper) collected from selected agricultural fields irrigated with canals fed IWW in Mingora city of Swat (Pakistan). Obtained results were compared with the tube well water irrigated soil and vegetables grown in it. Heavy metals accumulation was tested through wet digestion method and atomic absorption spectrophotometry (AAS). The metal transfer factor (MTF) of heavy metals from soil to vegetables was also determined along with the health index (HI) to assess the potential health risk of the metals towards consumers using Monte Carlo simulation technique. Analysis of water samples showed that the concentration in mg l^-1^ of heavy metals in IWW follows the trend Fe (6.72) > Cr (0.537) > Pb (0.393) > Co (0.204) > Mn (0.125) > Ni (0.121). Analysis of the soil samples irrigated with IWW followed the order of Fe (47.27) > Pb (2.92) > Cr (2.90) >Ni (1.02) > Mn (0.90) > Co (0.68) and Fe (17.12) > Pb (2.12) > Cr (2.03) >Ni (0.76) > Co (0.49) > Mn (0.23) irrigated with TWW. Heavy metals concentration values found in soil irrigated with IWW were higher than the soil irrigated with TWW. Similar trends were found for agricultural produces grown on soil irrigated with IWW and found higher than the normal allowable WHO limits, indicating higher possibilities of health risks if continuously consumed. MTF values were found higher than 1 for ladyfinger and green pepper for Pb intake and pumpkin for Mn intake. The current study suggests the continuous monitoring of soil, irrigation water and agricultural products to prevent heavy metals concentration beyond allowable limits, in the food chain. Thus, concrete preventive measures must be taken to reduce heavy metal accumulation through wastewater irrigation to protect both human and animal health in the study area of Mingora Swat Pakistan.

## 1. Introduction

Water is one of the necessities on earth. To survive, all plants and animals need water. Other uses of water apart from drinking include cooking, cleaning, recreation, farming, etc. Besides, for the healthy growth of agricultural produces, water is an essential part of the farm stock and manufacturing of different products as well. Several countries especially developing nations widely reuse wastewater for irrigation due to water scarcity and most often drop effluent discharge lines to natural drainage systems [1].

Wastewater contains toxic pollutants like heavy metals, whose long-term use can create problems for all living beings and degrades the quality of several agricultural produces as well. These toxic pollutants gradually accumulate in agricultural soil and consequently make their way into plants, making them potentially toxic for usage. Excess amounts of toxic pollutants accumulated in crops and/or vegetables grown in contaminated soil pose a serious threat to living beings who consume it. Vegetables, fruits, and cereals contain essential nutrients like carbohydrates, proteins, minerals, and vitamins required for good human health [2, 3]. However, the quality of vegetables which is the key factor, largely depend on well nutritious soil and groundwater, free from contaminants such as heavy metals. The most common heavy metals in industrial wastewater (IWW) include arsenic (As), cadmium (Cd), chromium (Cr), copper (Cu), zinc (Zn), nickel (Ni), manganese (Mn), lead (Pb), and iron (Fe). A very small amount of heavy metals such as cobalt (Co), Cu, Fe, Mn, molybdenum (Mo), vanadium (Vd), and Zn are required to run various biochemical and physiological functions for good health [2]. Nevertheless, an excess amount of these micronutrients is harmful for human, animals, and plants, and as a consequence trigger variety of adverse effects and diseases [4].

These heavy metals and contaminants originate from industries, municipal waste, and natural disasters [5, 6]. IWW contains a large proportion of toxic chemicals which is continuously being discharged to surface or groundwater and soil [7, 8]. Increased discharge of wastewater containing heavy metals in large quantities from industries is a matter of great concern for the survival of the ecosystem. Textile industry wastewater, when mixed with the irrigation water reduces seed development and initial growth of all vegetables and plants [3, 9]. Industrial applications such as smelting, pottery, paints, battery processing, arms manufacturing, and car industries are pouring various hazardeous heavy metals into the ecosystem especially Pb that trigger devastating effects.

Discharge of the untreated industrial effluents in the form of wastewater has been continuously increasing with the rapid and unplanned industrialization especially in developing countries. A large portion of the waste is dumped and discharged into nearby rivers and canals, which acts as a sink for the waste disposal of these industries [10, 11]. The heavy metals are continuously accumulating into the soil and being utilized for cultivation. Moreover, the use of such contaminated water for agricultural produces is a very common practice that possesses a great risk to human health [12]. Wastewater irrigation degrades the quality and growth of vegetables and crops thus creates various health issues as it contains an excess amount of heavy metals [12–15].

Continuous monitoring of irrigation water and agricultural produce is necessary. The heavy metals concentration in IWW can be minimized to an acceptable limit if treated before discharge. Soil absorbs these toxic chemicals and heavy metals and a result become harmful media for all agricultural produces like vegetables and other crops [16, 17]. The agricultural produce absorbs these heavy metals when soil becomes saturated with the heavy metals [18, 19].

Due to the shortage and unavailability of pure water, the agricultural water demands are fulfilled by the nearby available wastewater supplies at various places [20]. Such wastewater includes IWW coming out from industries as industrial effluent loaded with heavy metals including Fe, Cr, Pb, Ni, Co, and Mn. Consequently, untreated IWW causes major threats to the sustainability of the entire system including the environment, society, and economy. Society gets affected due to the consumption of polluted vegetables and suffers from various health issues and deterioration of overall quality of life. Consequently, three basic pillars of sustainability, environmental, social, and economic, are negatively affected because of using untreated IWW for agricultural purposes.

In developing countries like Pakistan, where the large population is living in the rural areas, are unaware of the potential hazards of heavy metals accumulation in the soil and agriculture produces grown on this soil. Although few studies have been conducted in relevance [21–24] that show the potential risk of affected soil and vegetables towards human health but no comprehensive study exists to date on the identification, characterization, and analysis of heavy metals (Fe, Pb, Cr, Ni, Mn, and Co) accumulation through wastewater irrigation in the study area. Therefore, the objective of this study was to investigate and carry out a quantitative analysis of the heavy metals accumulation in soil and selected local seasonal vegetables of the study area, Mingora, Swat (Pakistan). Besides, the metal transfer function (MTF) from soil to plant, an important factor of human disclosure has also been determined and presented. The hazard index (HI) associated with the daily intake of the accumulated heavy metals through contaminated vegetables has also been investigated using Monte Carlo simulation to estimate the potential human health risk due to their level of exposure.

## 2. Materials and methods

For heavy metal analysis, samples of industrial effluents and tube well were collected in clean dried bottles while vegetables and soil samples were collected in clean and airtight plastic bags. In all experiments, deionized water was used for sample preparation. Hydrochloric acid (33%), nitric acid (63%, Sigma Aldrich), and perchloric acid, used for digestion and treatment of the samples were of analytical grades. Water samples were collected from different locations of selected sampling sites (S1 Text in S1 File). The study area selected for the sampling was around effluent discharge channel where irrigation with wastewater has been a common practice. Local farmers use this industrial effluents mixed canal water for irrigation purposes. No specific permission was needed for collection of the water samples as all samples were collected from the open effluent discharge mixed canals. Soil and vegetables samples were collected from selected agricultural fields with the support of local farmers. The samples were taken from canals fed by industries and are termed as IWW. Each sample is a mixture of different collections from the sampling sites. Similarly, the sample of water is taken from the tube well of the study area that feeds the control sample soil and is termed as tube-well wastewater (TWW). Two different agricultural fields in the same study were chosen for the cultivation of similar agricultural produces grown with IWW fed canals and TWW, respectively. Geographically, the study area i.e. city of Mingora Swat, Pakistan, stretches between longitudes 72° 19ʹ 15ʺ to 72° 22ʹ 50ʺ E and latitude 34° 45ʹ 25ʺ to 34° 47ʹ 36ʺ N.

Four vegetable crops ladyfinger *(hibiscus esculentus)*, onion *(allium cepa)*, pumpkin *(cucurbita pepo and cucurbita moschata)*, and green pepper *(capsicum annum)* were selected for investigation purposes due to their large-scale seasonal cultivation and consumption in the agricultural fields of Mingora city Swat. The botanical information of these vegetables is given in SIF (S1 Table in S1 File).

Vegetable samples irrigated with IWW and TWW were collected from selected spots for comparison in the area at their same maturity stage and same seasons. Each sample of vegetables was scrubbed with distilled water to remove dirt and unwanted particles. The samples were first air-dried and then dried in an electric oven for 24 h at 80°C. Dried samples were further dried for 24 h at 100°C. The samples were converted into powdered form (80 mesh) using a commercial mixer (TSK- WestPoint, France) and kept in plastic bags for acid digestion. In the wet sample preparation process, 0.5 g of the sample was mixed with an already prepared solution of 1 mL perchloric acid and 4 mL nitric acid (1:4) in acid digestion. After cooling, the samples were filtered. Deionized water was added to the filtrate to have a final volume of 25 mL.

The soil samples were dried in the oven to remove the water. Aqua regia of HNO_3_ and HCl (1:3) were prepared in the round bottom flask. 2 g of each sample was mixed with 10 mL of aqua regia solution. Then, the samples were heated in an oven at 90°C for 20–25 min. After cooling, the samples were filtered using Whatman 42 filter paper. The filtrate was diluted with distilled water using a volumetric flask to fill up to the required volume for analysis purposes. All the samples were prepared using the same procedure.

### 2.1 Analysis and characterization

All samples of IWW, soil, and vegetables of the study area were analyzed for selected heavy metals (Fe, Pb, Cr, Ni, Mn, and Co) using Atomic Absorption Spectrophotometer (Perkin Elmer AAS-700) using standard digestion procedure as reported in the literature [25]. Samples were analyzed three times and the mean values were recorded for statistical purposes and comparison.

MTF from soil to plant is one of the main factors of human exposure, which describes the transfer of metal from soil to plant body. The MTF was calculated by the following Eq (1) [26]:

$$MTF=C_{plants}÷C_{soil}$$ (1)

where *C*_*plant*_ and *C*_*soil*_ is the concentration of heavy metal in plant and soil, respectively. It helps to assess the potential ability of agricultural produce to transfer heavy metals from soil to their edible parts and explains the absorption phenomenon of heavy metals transfer from the contaminated soil [27, 28].

To assess the human health risk of heavy metals, it is necessary to calculate the level of human exposure to that metal by following the route of exposure of pollutants to the human body. To evaluate the level of health hazard, *HI* was calculated using (Eq 2).


$$HI=\sum_{i=1}^{n}TQM$$ (2)

*HI* of a specific heavy metal determines the level of its risk that the consumer is facing. Value of *HI* > 1 for any metal in food crops means that the consumer population faces a health risk. Value of *HI* < 1 for any heavy metal represents that the subject crop in the food chain is safe to consume [26, 27]. *Target hazard quotients (THQ)* refers to the ratio of the daily intake of metals (EDI, *mg metal/kg body weight/day*) in the agricultural produces (vegetables, crops, fruits) to the oral reference dose (*RfD*). *THQ* was calculated from Eq (3).


THQ=EDI÷RfD (3)

*Rf D* (mg/kg bw/day) is the reference dose values of the targeted metals. The R*f*D values for Fe, Pb, Ni, Cr, Mn, and Co are 0.7, 0.004, 0.02, 0.1.5, 0.033, 0.090 mg/kg bw/day, respectively are given in the literature [29]. *EDI* was calculated using Eq (4).

$$EDI=\frac{Cmetal\times Cf\times EF\times ED\times IR}{BW\times AT}$$ (4)

where *C*_*metal*_, *Cf*, *EF*, *ED*, *IR*, *BW*, *and AT* represent the heavy metal concentrations in plants (mg/kg), conversion of the fresh to dry weight (0.085), exposure frequency to the trace element (day/year), the exposure duration (year), the food ingestion rate per day for the respective food item (kg/day), the reference bodyweight (kg), and the averaged exposure time (day), respectively [30].

In another way, for probabilistic simulation of HI related to metals, in this study, Monte Carlo simulation was used [31]. For probabilistic risk assessment modeling, the Monte Carlo simulation is one of the most broadly used approaches that can evaluate the variability and uncertainty in the several parameters of the human health risk assessment procedure [32]. A simplified approach to performing Monte Carlo simulation is to create the model without uncertainty in Microsoft Excel software, then use the spreadsheet-based application, such as using Crystal Ball (11.1.2.4) software [31, 32]. The calculation process is repeated several times and estimates the average, minimum, maximum, standard deviation, percentiles, and some other statistical indicators as the final results. Therefore, the results obtained from the Monte Carlo simulation technique are more reliable and more valuable compared with the point estimate method [33]. In the present work, the variability and sensitivity analysis of the predictions of the risk assessment model was carried out using the Monte Carlo simulation technique. The description of input parameters into the Monte Carlo simulation is summarized in Table 1.

**Table 1:** Distributions of parameters for different population groups used in risk assessment model with Monte Carlo simulation.

Parameters	Unit	Distribution	Children	Adults	Ref.
*EDI*	mg/kg/day	-	Calculated using Eq (4)		-
*THQ*	-	-	Calculated using Eq (3)		-
*HI*	-	-	Calculated using Eq (2)		-
*C*_*metal*_	mg/kg	Log normal	Varied according to metals		-
*IR*	kg/day	Normal	IR of ladyfinger: 0.0155 ± 0.01	IR of lady finger: 0.058 ± 0.01	[34, 35]
			IR of Pumpkin: 0.045 ± 0.01	IR of Pumpkin: 0.0648 ±0.01	
			IR of Onion: 0.0058 ± 0.01	IR of Onion: 0.0218 ±0.01	
			IR of Green pepper: 0.0058 ± 0.01	IR of green pepper: 0.0218 ±0.01	
*EF*	day/year	Triangular	Min:180	Min:180	[34]
			Mode:345	Mode:345	
			Max: 365	Max: 365	
*ED*	Year	Uniform	1–7	26–70	[36]
*BW*	kg	Log normal	32.7	73.5	
*AT*	Days	Fixed value	32.7	73	[37]
*RfD *	mg/kg/day	Fixed value	Varied according to metals		[29]

To perform sensitivity analysis, the Monte Carlo simulation was run in different iterations and chooses the values of the parameters from their distribution fitted to input data and consequently calculates both point value and the distribution of exposure and risk. After developing a deterministic model, you replace point estimates with probability distribution assumptions and forecast the distribution of the output. The forecasted output distribution is used to assess the riskiness of the situation [33, 38]. An uncertainty analysis based on Spearman rank-order correlation was employed to understand how uncertainty and variability of input parameters can influence on uncertainty of HI as response variables in the models. In the present study, the Monte Carlo simulation was run for 5000, 10,000, and 50,000 iterations. Results were compared from independent simulations to test the convergence and stability of the results for the highly skewed distributions (data are not shown). For 5000 iterations, the estimated means, standard deviations, variances, and the 90^th^ and 95^th^ percentiles do not agree within 1%. For two independent runs of 10,000 and 50,000 iterations, all the summary statistics agreed within 1%. From this, we conclude that 10,000 iterations are sufficient to ensure convergence and stability of the output distributions. In addition, Monte Carlo simulation with 10,000 iterations takes around 6 min.

## 3. Results and discussion

## 3.1 Water analysis for heavy metals

Water samples collected from the sampling site (S1 Text in S1 File) were analyzed using the standard analytical method as reported in the literature [39]. Results were compared with the World Health Organization (WHO) recommended limits of selected heavy metals that were retrieved from the literature as shown in Table 2.

**Table 2:** Concentrations of heavy metals in IWW and TWW in comparison with WHO limits [26, 40, 41].

Source of Heavy Metals (mg.l^-1^)	Fe	Pb	Cr	Ni	Mn	Co
IWW	6.72±0.023	0.393±0.13	0.537±0.01	0.121±0.01	0.125±0.02	0.204±0.03
TWW	1.17±0.078	0.043±0.014	0.416±0.05	0.091±0.01	0.033±0.01	0.116±0.02
WHO limit	5.00	0.01	0.05	0.07	0.20	0.025

The order of heavy metals concentration in IWW follows as Fe>Cr>Pb> Co>Mn>Ni as shown in Table 2. Fe and Cr concentrations are the highest as compared to other heavy metals. For example, the value of Fe is 6.72± 0.023 mg.l^-1^ which is much greater than the TWW of 1.17±0.078 mg.l^-1^ and exceed the WHO allowable limit of 5 mg.l^-1^. Pb concentration is 0.393±0.13 mg.l^-1^, which is also above the WHO permissible limit of 0.01 mg.l^-1^. Similarly, Co also exceeds the WHO allowable limit of 0.025 mg.l^-1^. As evident from **Table 2**, the heavy metals concentration is higher in IWW as compared to that in TWW. This is because the polluted water from IWW is directly used for irrigation purposes to grow crops and vegetables. Consequently, it affects the quality of water, crops, and vegetables. In addition to that, it is noted that the selected IWW of selected locations in Mingora city has the highest concentration of heavy metals as compared that in other areas due to the existence of different small and medium scale industries Khan *et al*. [42] conducted a similar study in District Charsadda and found Fe concentration of 0.01, Pb of 0.0075, Ni of 0.002 and Cr of 0.005 mg.l^-1^. Similarly, Muhammad *et al*. [43] reported the heavy metals concentration as Pb 0.009, Ni 0.004 and Cr 0.007 mg.l^**-1**^ in district Kohistan wastewater. Khanoranga and Khalid [44] in a similar study investigated and assess the quality of groundwater in the Baluchistan province of Pakistan and found elevated concentrations of heavy metals with a low water quality index (WQI). Titilawo *et al*. [40] reported in a similar study concentration of heavy metals as Fe = 0.2 and Cr = 0.19 mg.l^-1^. Since the water samples in this study contains a higher concentration of heavy metals due to the different industries in the area and the soil ability to absorb these heavy metals, it will cause problems like failure of kidneys, loss of bones, liver damage, and high blood pressure and other serious health hazards.

In the study area (Mingora city), IWW is directly used for irrigation purposes without treatment. Therefore, continuous discharge of wastewater leads to enrichment of heavy metals in the soil as shown in Table 3. Therefore, soil samples from the field fed by the IWW and TWW termed as SIWW and STWW, respectively, were taken and analyzed for the selected heavy metals.

**Table 3:** Concentrations of heavy metals in soil irrigated with IWW and TWW.

Heavy Metals (mg.l^-1^)	Fe	Pb	Cr	Ni	Mn	Co
IWW	47.27±0.12	2.92±0.10	2.90±0.04	1.02±0.02	0.90±0.03	0.68±0.05
TWW	17.12±0.27	2.12±0.21	2.03±0.03	0.76 ±0.04	0.23±0.01	0.49±0.01

## 3.2 Soil analysis for heavy metals

It is very clear from Table 3 that the Fe concentration is the highest in soil samples followed by Pb>Cr>Ni>Mn>Co which exceeds the WHO allowable limits reported in the literature [40, 41]. This increase in the concentration of heavy metals in soil is mainly due to the irrigation by untreated IWW, which strongly affects the quality of soil and thus, vegetables as shown in Table 4.

**Table 4:** Concentrations of heavy metals in vegetables irrigated with IWW.

Heavy Metals (mg.l^-1^)	Fe	Pb	Cr	Ni	Mn	Co
Ladyfinger	8.09±0.06	3.51±0.27	0.43±0.04	0.22±0.05	0.21±0.01	0.09±0.05
Pumpkin	24.90±0.18	1.61±0.21	0.51±0.03	0.39±0.04	0.97±0.01	0.07±0.01
Onion	3.60±0.04	0.67±0.03	0.44±0.06	0.25±0.04	0.16±0.02	0.14±0.06
Green pepper	1.70±0.04	3.58±0.16	2.30±0.06	0.20±0.06	0.08±0.03	0.10±0.01

More specifically, high Pb values are linked with bad sanitation, vehicular emissions, and Pb-based batteries while the main sources of Cd are chemical and electroplating industries, agricultural activities, sewage water, and vehicular emissions [45]. Furthermore, the textile industry is mainly responsible for heavy metals such as Cd, Cu, and Fe in the area [46]. Reemtsma *et al*. [47] reported that Fe, Pb, and Zn originate from urban runoff, while Cr and most alkaline earth metals are provided by municipal wastewater. To summarize the potential source of heavy metals is agricultural and industrial activities. In addition, air pollution could be related to surface runoff mainly due to rainwater.

## 3.3 Analysis of agricultural products for heavy metals and their impacts

Table 4 shows that the concentration of heavy metals in all vegetables analyzed is higher than the WHO allowable limits. Thus, it can be concluded that IWW not only affects the soil quality (Table 3) but also the growth of vegetables irrigated with IWW (Table 4).

To compare these results with vegetables irrigated with TWW (as a control sample), vegetable samples irrigated with TWW were analyzed as shown in Table 5.

**Table 5:** Concentrations of heavy metals (mg.l^-1^) in vegetables irrigated with TWW.

Heavy Metals (mg.l^-1^)	Fe	Pb	Cr	Ni	Mn	Co
Ladyfinger	2.22±0.10	0.73±0.11	0.39±0.04	0.18±0.18	0.16±0.02	0.04±0.02
Pumpkin	3.13±0.18	0.91±0.06	0.53±0.10	0.27±0.04	0.42±0.04	0.01±0.02
Onion	2.16±0.11	0.74±0.04	0.45±0.06	0.24±0.02	0.28±0.02	0.06±0.05
Green pepper	0.78±0.10	0.69±0.07	0.60±0.04	0.150±0.03	0.08±0.01	0.12±0.04

Comparative analysis of the investigated samples, irrigated with TWW in Table 5 shows that the heavy metal concentrations are very low as compared to the results obtained in Table 4. Lower values of heavy metals accumulation proves that the accumulation in IWW irrigated samples (vegetables) is largely due to the presence of heavy metals in IWW. Concentrations of Fe, Ni, and Mn are highest in Pumpkin compared to other vegetable samples irrigated by TWW. Similarly, green pepper contains the highest concentration of Pb while Co concentration is highest in onion. Furthermore, Fe concentration is lowest in green pepper than other vegetables while the onion is more resistive to Pb and Green pepper and Ladyfinger to Ni. Similarly, Co concentration is negligible in green pepper while green pepper is more susceptible to lead and Cr.

### 3.4 MTF analysis

**Fig 1:**
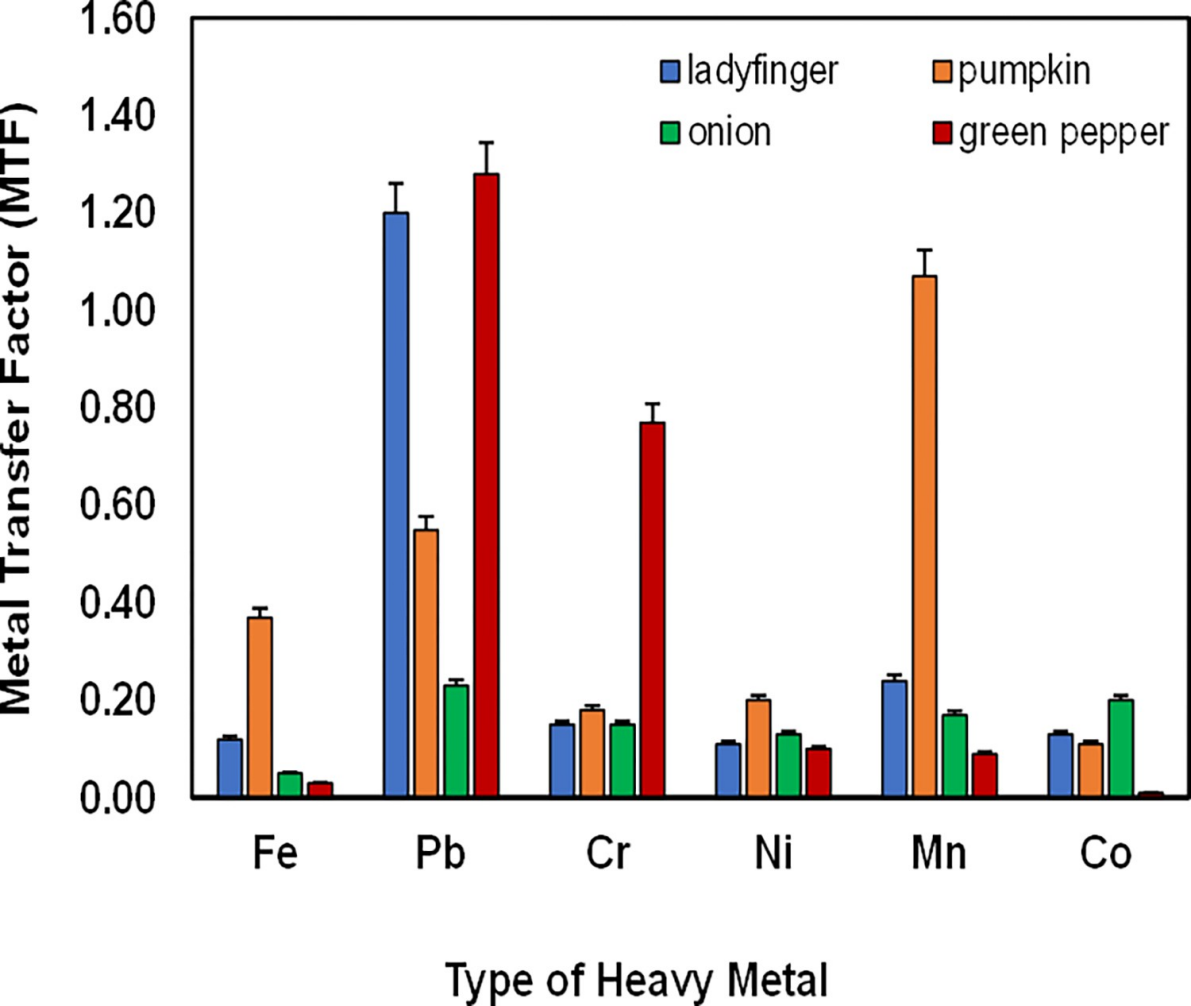
MTF of Fe, Pb, Cr, Ni, Mn, and Co from soil into vegetables. MTF is calculated from the mean values of three samples of each heavy metal.

To investigate the concentrations of various heavy metals in selected vegetable samples, it is necessary to evaluate their MTF. The MTF explains the absorption phenomenon of heavy metals into vegetable samples from the contaminated soil [28, 47]. By analyzing the MTF, the extent of heavy metals accumulation transferred from contaminated soil to vegetables can be determined. MTF of ladyfinger, onion, pumpkin and green pepper were determined as shown in Fig 1.

The sequence of MTF decreased in the order of Pb>Mn >Cr>Co>Fe>Ni in ladyfinger. Similarly, the MTF of pumpkin follows Mn>Pb>Fe>Ni >Cr>Co. The mean MTF of Pb was highest (0.227) in onion while Fe has the lowest MTF (0.053). Overall absorption sequence of metals in onion follows Pb>Co>Mn>Cr>Ni>Fe. Similarly, data obtained showed that MTF was decreased in the order of Pb>Cr>Ni>Fe>Mn>Co in green pepper with the highest and lowest MTF of 1.28 and 0.007, respectively.

**Fig 1** indicates that lady finger has the maximum MTF for Pb while Pumpkin for Fe and Mn. Similarly, green pepper has the maximum MTF for Pb and Cr and the minimum for Fe and Co. Onion while having the maximum MTF for Co. The MTF for Fe in pumpkin is higher compared to other vegetables. Furthermore, lady finger and green peppers are found to show comparatively a higher MTF amongst the studied vegetables **(Fig 1)** while onion has the least MTF. Our studies show that the accumulation of heavy metals in investigated vegetables varies. In addition, vegetables with the highest MTF reveal their behavior towards heavy metal intake from the soil [27]. The highest MTF in vegetables could be due to the highest amount already present in these vegetables. However, this theory cannot be considered and the reason being is these samples irrigated with TWW samples have already been investigated and have been found less as compared to IWW irrigated samples **(Table 5).**

### 3.5 HI analysis

**Fig 2:**
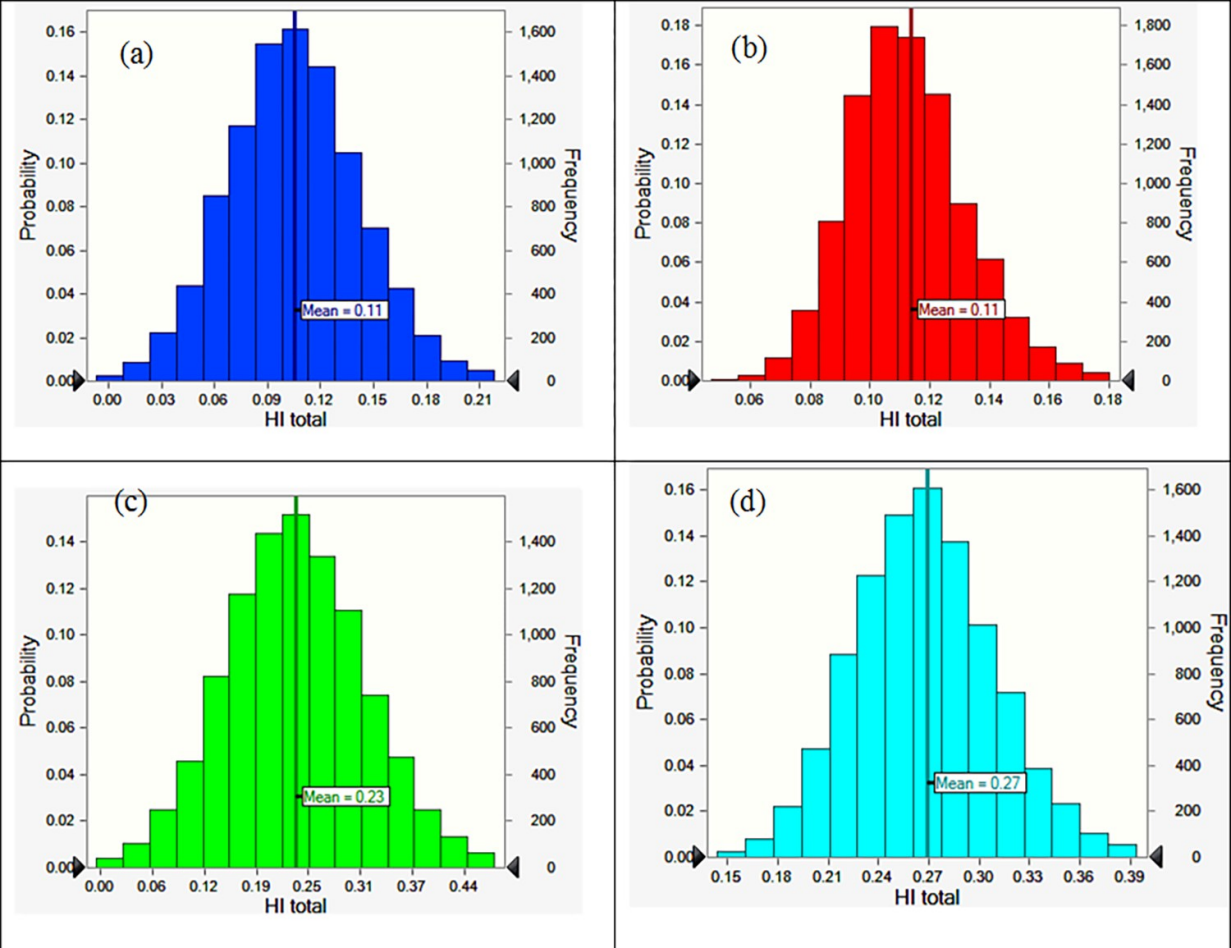
Cumulative distribution of the HI total of heavy metals for (a) vegetables irrigated with TWW for children, (b) vegetables irrigated with TWW for adults, (c) vegetables irrigated with IWW for children, and (d) vegetables irrigated with IWW for adults.

By considering the probabilistic approach in the simulation of HI total by Monte Carlo simulation, the mean values of the distributions of the HI for different population groups are shown in Fig 2.

Overall, the results show that adults’ exposure is higher than children which also indicates their higher health risk as compared to adults. As displayed in Fig 2, the mean value of HI total was 0.11, 0.11, 0.23, and 0.27 for vegetables irrigated with TWW for children and adults and vegetables irrigated with IWW for children and adults, respectively. The mean values of HI total are lower than 1 and ascribed no health risks for both groups of populations.

**Fig 3:**
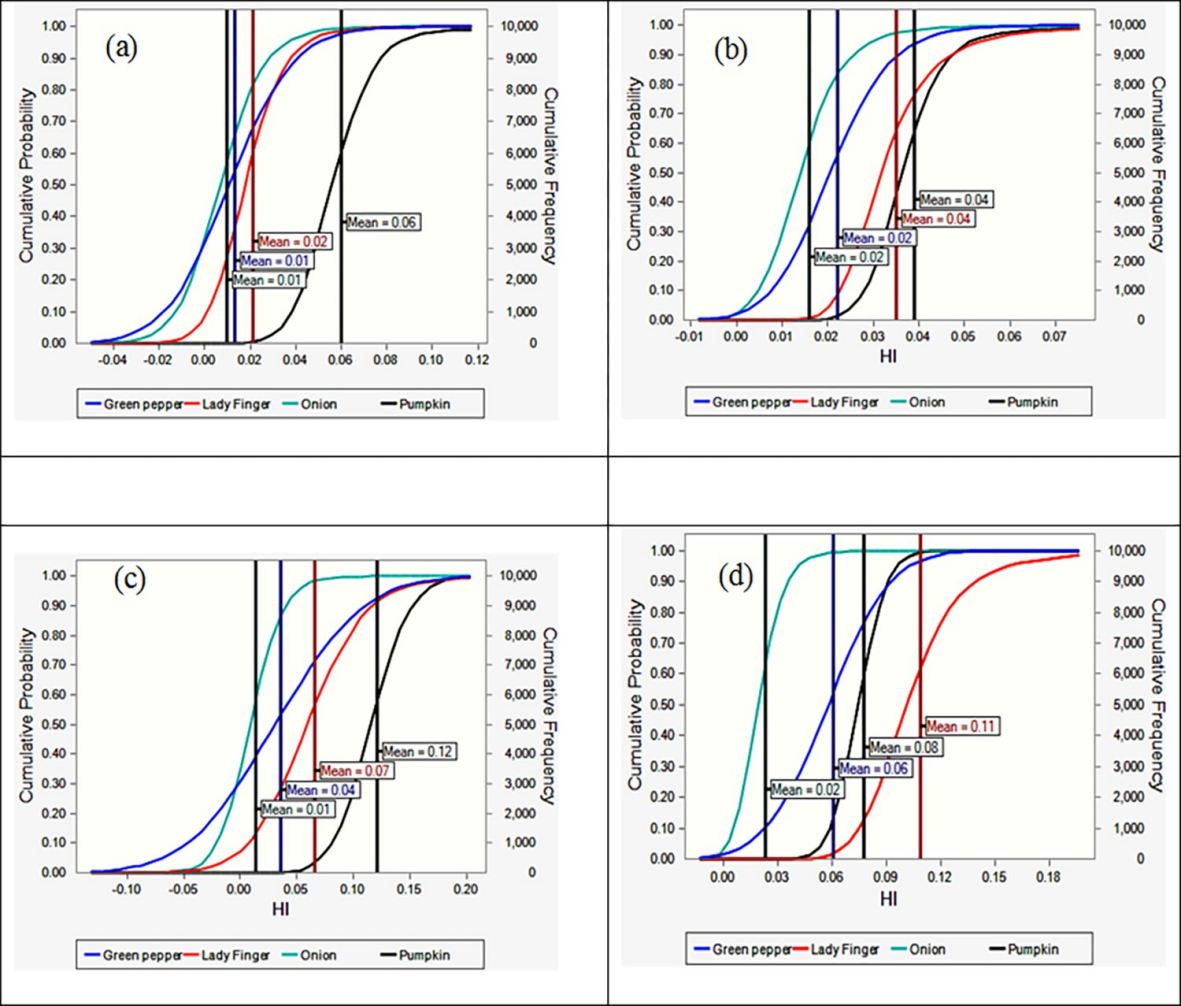
Cumulative probability of HI as a forecast of vegetables for (a) vegetables irrigated with TWW for children, (b) vegetables irrigated with TWW for adults, (c) vegetables irrigated with IWW for children, and (d) vegetables irrigated with IWW for adults.

By considering groups of the population, the HI was computed based on the vegetable type and depicted in Fig 3.

When TWW was used for vegetable irrigation, for both population groups, the mean value of HI of pumpkin is higher than other vegetables. But, in the case of IWW irrigation, the highest mean value of HI was related to pumpkin and lady finger for children and adults, respectively.

**Fig 4:**
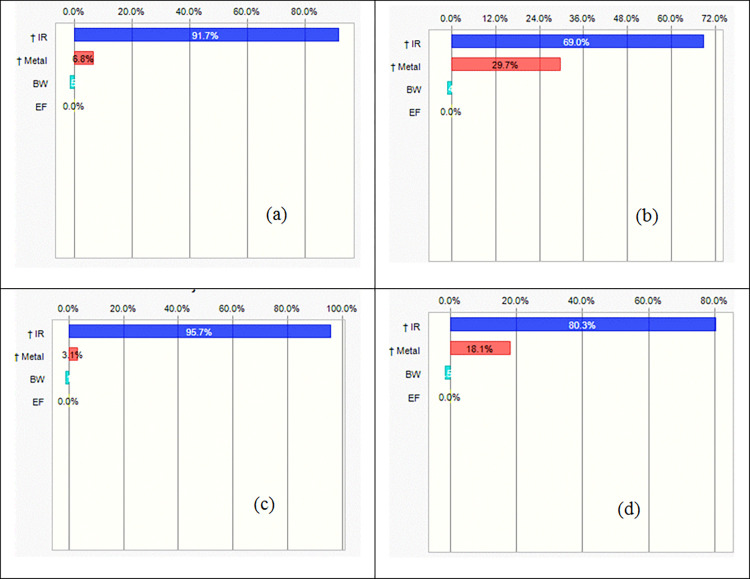
Sensitivity analysis of HI total for (a) vegetables irrigated with TWW for children, (b) vegetables irrigated with TWW for adults, (c) vegetables irrigated with IWW for children, and (d) vegetables irrigated with IWW for adults.

To determine the effect of input parameters on HI total, the sensitivity analysis was conducted and showed that the changing of a unit in the values of input parameters, leads to how much change in the final result [31]. In the case of both water sources, the results of sensitivity analysis for children and adults are shown in Fig 4.

Overall, the results of sensitivity analysis indicated that the ingestion rate (IR) is the main influencing parameter on the HI total for both studied groups of population (contribution > 69%) the result agrees with the results reported by Sanaei *et al*. [31]

## 4. Conclusions

Concentrations of heavy metals in IWW and their effects on soil and agricultural produce in comparison with TWW in Mingora, Swat was investigated.

- Sample analysis showed that the concentration of heavy metals in IWW irrigated soil and vegetables is higher than the TWW and exceeds the WHO limits.
- The concentration of heavy metals in wastewater follows the order of Fe >Cr>Pb> Ni> Co> Mn. Specifically, the Fe, Pb, Cr, Ni concentrations were 6.72±0.02, 0.39±0.13, 0.54±0.01, 0.12±0.01 mg.l^**-1**^, respectively. Mn concentration was 0.13±0.02 mg.l^**-1**^ is well below then the WHO allowable limit of 0.50 mg.l^**-1**^.
- Pb and Fe have relatively higher concentrations in the soil as well as in vegetables that greatly affect the quality of vegetables.
- Fe and Pb have the highest MTF in pumpkin and lady finger.
- The HI values of metals in the case of adults are higher than children indicating that contamination in most of the vegetables is a greater threat to adults in the studied area with long-term uses of wastewater for irrigation.

## Supporting information

S1 File (DOCX)
