# Supplementary material for: Assessment of heavy metals accumulation in agricultural soil, vegetables and associated health risks
Source: PLoS One. 2022 Jun 16;17(6):e0267719. doi: 10.1371/journal.pone.0267719 (PMC9202934; doi:10.1371/journal.pone.0267719)
Supplement: S1 File — (DOCX) [file pone.0267719.s001.docx]

**Assessment of heavy metals in wastewater and its impact on agricultural soil and vegetables: characterization and analysis**

Nehar Ullah^1^, Maqsood Ur Rehman^1^, Bashir Ahmad^2^, Irshad Ali^1^, Muhammad Younas^1*^, Muhammad Sagheer Aslam^3^, Atta-ur Rahman^4^, Ensiyeh Taheri^5,6^, Ali Fatehizadeh^5,6^, Mashallah Rezakazemi^7,*^

^1^ Department of Chemical Engineering, Faculty of Mechanical, Chemical and Industrial Engineering, University of Engineering & Technology, Peshawar, Pakistan

1. Department of Plant Protection, Agricultural University, Peshawar, Pakistan
2. National Institute of Urban Infrastructure and Planning, University of Engineering & Technology, Peshawar, Pakistan
3. Department of Geography, University of Peshawar, Peshawar, Pakistan
4. Environment Research Center, Research Institute for Primordial Prevention of Non-communicable Disease, Isfahan University of Medical Sciences, Isfahan, Iran
5. Department of Environmental Health Engineering, School of Health, Isfahan University of Medical Sciences, Isfahan, Iran
6. Faculty of Chemical and Materials Engineering, Shahrood University of Technology, Shahrood, Iran

* Corresponding author: [*m.younas@uetpeshawar.edu.pk*](mailto:m.younas@uetpeshawar.edu.pk)*,* [*mashalah.rezakazemi@gmail.com*](mailto:mashalah.rezakazemi@gmail.com)

**Text S1. Study area**

Mingora, the capital city of District Swat, located at an average altitude of 984 m (above sea level) in the northwestern part of Pakistan. Geographically, the city of Mingora stretches between longitudes 72̊ 19ʹ 15ʺ to 72̊ 22ʹ 50ʺ E and latitude 34̊ 45ʹ 25ʺ to 34̊ 47ʹ 36ʺ N. Relatively, it is surrounded by lush green mountainous ranges of Hindukush in North-South and canals taken-out from river Swat. District Swat has an area of 5 337 km^2^ and has a population of about 1.3 million with a density of 230/km^2^ [1].

In northern part of the province, Mingora is the most populous and rapidly growing city. During past few decades, the population of Mingora city has increased at an exponential rate, as it was inhabited by 15,920 persons in 1961, which gradually increased to 38,499 (1972), 88,078 (1981), 173,868 (1998) and marked the figure of 331,091 in 2017. During 1998 to 2017 population censuses, the population of Mingora city grew at an average annual growth rate of 4.7%.

Being a divisional headquarter, Mingora city is well connected with rest of the country. It offers extensive services to people living in districts of Dir Upper, Dir Lower, Chitral, Swat, Buner, Malakand, Shangla and adjoining areas. The climate is continental type with hot/warm summer and cool winter. The average annual temperature in Mingora is 19.3 ̊C. November is the driest month (22 mm), while August receives the highest average monthly rainfall of 134 mm [2]. Rainfall is mostly received during spring, winter and summer monsoon. Topographically, the slope of the study area is mainly from south to north. The remote sensing-based land use land cover analysis revealed that Mingora city is surrounded by fertile food basket land dominated by vegetables, orchards and cereal crops.

The rationale behind these locations is the existence of medium scale industries including plastic, paper, chemicals, rubber, pharma, cosmetics, textiles, and steel, which discharges huge amount of industrial wastewater directly into water bodies containing heavy metals. In the sub-urb of Mingora city, the agricultural land is irrigated with these wastewater discharge. Samples of water, soil and largely consumed vegetables like ladyfinger, pumpkin, onion, and green pepper were collected from the farmland of selected locations. The botanical information of these vegetables is given in **Table S1**.

**Table S1: Details of vegetable samples investigated [3, 4].**

| **Agricultural produce** | **Botanical Name** | **Botanical Family** | **Edible Parts** | **Grow in: *(Soil)*** |
| --- | --- | --- | --- | --- |
| Ladyfinger | Hibiscus esculentus | Molvaceae | Fruits, seeds | Grow well in well-drained soil, rich in organic matter but ideal soil is sandy loam to clay loam |
| Pumpkin | Cucurbita pepo and Cucurbita moschata | Cucurbitaceae | Fruits, seeds, flowers | Well drained fertile loamy soil |
| Onion | Allium cepa | Amaryllidaceae | Leaves, fruits | Grow in almost any soil from sandy loams to heavy clay |
| Green pepper | Capsicum annum | Solanaceae | Fruits, seeds | Well-drained warm, sandy or loamy soil. |

All the samples were placed in standard water bottles and clean zipper-type polyethylene bags to avoid any contamination and digestion. The sealed samples were immediately transferred to laboratory for further investigation.

**References**

1. Sher, H., et al., *Economic benefits of high value medicinal plants to Pakistani communities: an analysis of current practice and potential.* Journal of Ethnobiology and Ethnomedicine, 2014. **10**(1): p. 71.

2. Imran, S., L. Bukhari, and S. Gul, *Water Quality Assessment Report: Mingora City District Swat Khyber Pakhtunkhwa 2018. )*. 2018, Pakistan Council of Research in Water Resources (PCRWR. p. 40.

3. Malik, M.N., E.L. Bashir, and R. Bantel, *Horticulture*. 1994.

4. Gupta, N., et al., *Trace elements in soil-vegetables interface: Translocation, bioaccumulation, toxicity and amelioration - A review.* Sci Total Environ, 2019. **651**(Pt 2): p. 2927-2942.
